# MBD2 upregulates miR-301a-5p to induce kidney cell apoptosis during vancomycin-induced AKI

**DOI:** 10.1038/cddis.2017.509

**Published:** 2017-10-12

**Authors:** Juan Wang, Huiling Li, Shuangfa Qiu, Zheng Dong, Xudong Xiang, Dongshan Zhang

**Affiliations:** 1 Department of Emergency Medicine, Second Xiangya Hospital, Central South University, Changsha, Hunan, People’s Republic of China; 2 Emergency Medicine and Difficult Diseases Institute, Second Xiangya Hospital, Central South University, Changsha, Hunan, People’s Republic of China; 3 Department of Ophthalmology, Second Xiangya Hospital, Central South University, Changsha, Hunan, People’s Republic of China; 4 Department of Nephrology, Second Xiangya Hospital, Central South University, Changsha, Hunan, People’s Republic of China; 5 Department of Cellular Biology and Anatomy, Medical College of Georgia at Georgia Regents University and Charlie Norwood VA Medical Center, Augusta, GA, USA

## Abstract

Despite DNA methylation occurred in acute kidney injury (AKI), how it influenced progression of AKI remains unclear. Methyl-CpG-binding domain protein 2 (MBD2), a protein readers of methylation, was used to analyze the impact of DNA methylation on vancomycin (VAN)-induced AKI. Here, in cultured human kidney tubular epithelial cells (HK-2), we show that knockdown of MBD2 by siRNA attenuated VAN-induced apoptosis, caspase activity, and the expression of BAX and cleaved caspase 3. Interestingly, knockdown of MBD2 by siRNA was associated with the suppression of miR-301a-5p. Mechanistic studies confirmed MBD2 binds to these methylated CpG elements of miR-301a-5p promoter, and then activates miR-301a-5p promoter by suppressing methylation. Furthermore, anti-miR-301a-5p significantly blocked VAN-induced apoptosis and caspase activity in HK-2 cells, which was accompanied by downregulation of p53, and upregulation of MITF, HDGF and MDM-4 together. The latter genes were further identified as target genes of miR-301a-5p, and silencing of MDM-4 promoted p53 accumulation. *In vivo*, mice with MBD2 knockout (MBD2-KO) were counteracted to VAN-induced AKI, indicated by the analysis of renal function, histology, apoptosis and inflammation. MBD2-KO also significantly suppressed the expression of miR-301a-5p, p53, BAX and cleaved caspase 3, and restored the expression of MDM-4, MITF and HDGF. Finally, *in vivo* inhibition of miR-301a-5p also ameliorated VAN-induced AKI. Together, these results show the novel MBD2/miR-301a-5p/MITF, HDGF and MDM-4/p53 pathway in VAN-induced AKI.

Vancomycin (VAN) is one of the most commonly potent and most used glycopeptide antibiotic for treatment of Staphylococcus epidermidis, and methicillin-resistant Staphylococcus aureus infection (MRSA).^1, 2^ The recent study confirmed that prevalence of MRSA with reduced susceptibility to VAN was gradually increased, which prompted specialists to recommend for higher target trough serum concentrations.^3, 4, 5^ However, the use of larger doses of VAN has led to a wider report of acute kidney injury (AKI) cases.^6, 7^ Although the most of them are mild or even reversible, both the greater incidence of end-stage renal disease (ESRD) and higher mortality rate may be associated with their germination.^8, 9, 10, 11^ These data further prompted us to renewed concern about the molecular mechanism of VAN-induced AKI. Although previous results revealed that proinflammatory oxidation, mitochondrial dysfunction and cellular apoptosis involved in VAN-induced AKI,^6, 12, 13, 14, 15, 16, 17^ the underlying mechanisms are still largely unknown.

DNA methylation, a major epigenetic inheritance mechanism, represents outcome of environmental insults interacted with different kinds of cells.^18^ DNA methylation has an important role in epigenetic gene modulation during development and diseases including cancer and AKI.^19, 20, 21^ The protein methyl-CpG-binding domain (MBD) family (MBD1-4, and MeCP2), as protein readers of methylation, actively involves in DNA methylation–mediated transcriptional repression and/or heterochromatin formation and is liable for maintaining and interacting with DNA methylome.^22^ Furthermore, MBD protein 2 (MBD2) has been linked to disease such as immune system function and tumorigenesis.^23, 24, 25, 26^ A recent study reported that inhibition of MBD2 protected against mice hind-limb ischemic injury by suppression of endothelial cells apoptosis.^22^ Although MBD2 is high expressed in normal kidney tissues,^26^ little is known about its functions in kidney disease.

In view of these findings, this study was initiated to assess whether inhibition of MBD2 may block VAN-mediated AKI by using genetic inhibitory approaches. Moreover, we have investigated the mechanism whereby MBD2 contributed to renal injury. We for the first time demonstrate that blockade of MBD2 leads to the attenuation of VAN-mediated AKI. We further show that MBD2 may induce miR-301a-5p to suppress anti-apoptosis genes including HDGF and MITF, and also inhibit MDM-4 for p53 activation, resulting in renal cell apoptosis and subsequent renal injury.

## Results

### VAN induced the expression of MBD2 in HK-2 cells and mice kidneys

**Figure 1:**
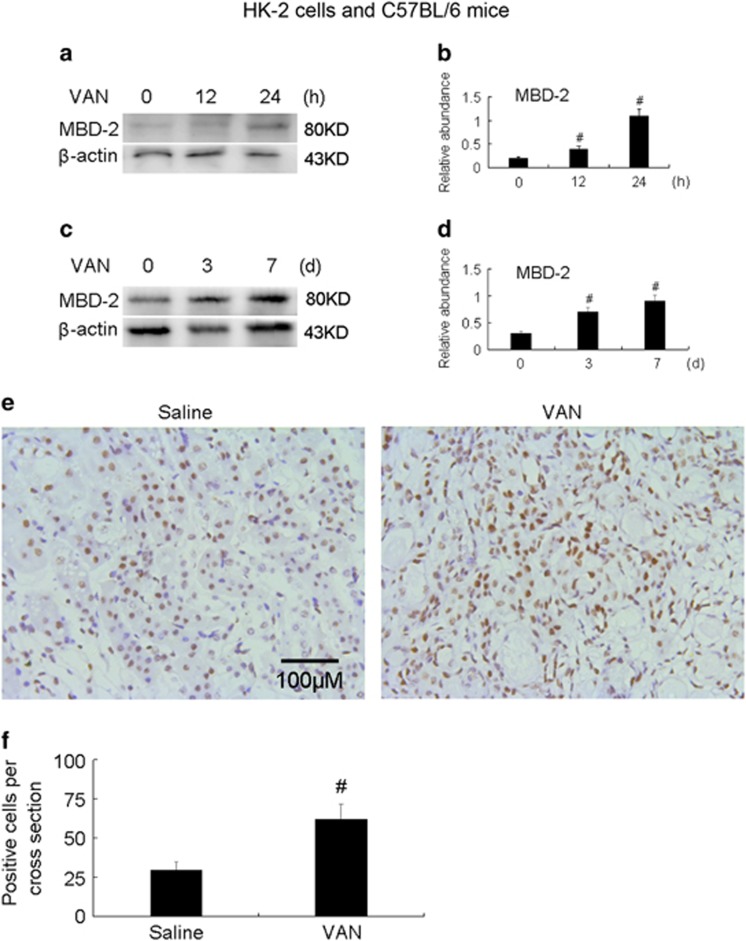
MBD2 is induced by VAN in HK-2 cells and mice nephrotoxic AKI. HK-2 cells were treated with 4 mm/l VAN for 0–24 h. Male C57BL/6 mice were (**a**-**f**) injected with 600 mg/kg VAN (*n*=8) for 0–7 days of examination. In **a** and **b**, cell lysates were analyzed by immunoblot with MBD2 and *β*-actin at the indicated time points (loading control). In **c**–**f**, kidneys were harvested for immunoblot analysis of MBD2, *β*-actin and immunohistochemistry staining of MBD2 at the indicated time points. *β*-Actin as an internal control. Scale bar, 100 μM. Data were expressed as means±S.D.; ^#^*P<0.05* *versus* day 0 or Saline group. Original magnification, × 200. Data are the representative of at least four separate experiments

We first investigated whether VAN induced the expression of MBD2 in VAN nephrotoxic AKI. MBD2 is induced gradually in HK-2 cells and kidneys at the indicated time points (Figures 1a–d). Furthermore, the immunohistochemical staining results showed that MBD2 is expressed mainly in the nuclei of the tubular cells (Figures 1e and f). These data for the first time indicate the induction of MBD2 in VAN nephrotoxic AKI.

### MBD2 siRNA suppressed VAN-induced HK-2 cells apoptosis

**Figure 2:**
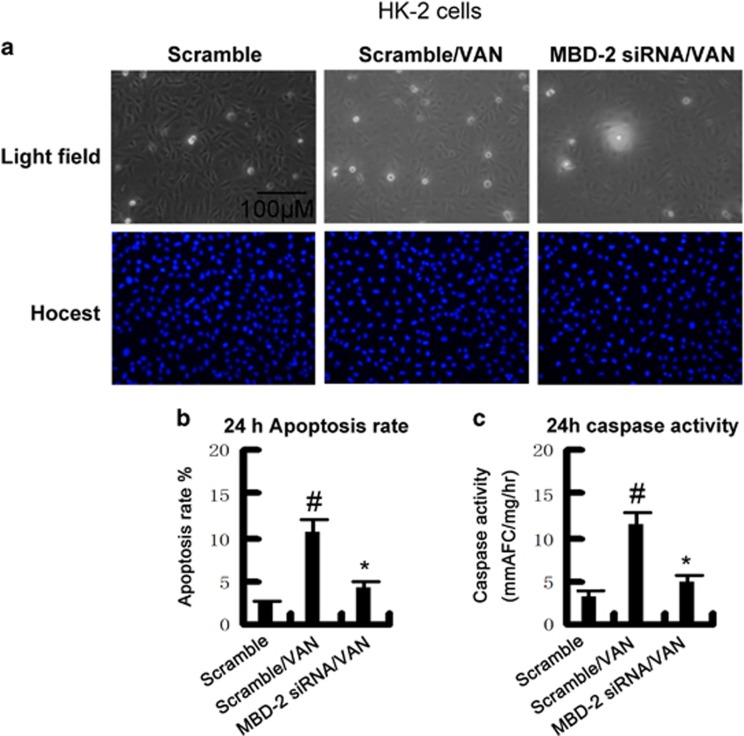
Effects of siRNA MBD2 on VAN-induced apoptosis in HK-2 cells. HK-2 cells were transfected with 50 nmol/l MBD2 siRNA or the scrambled sequence (scramble). Cells were then left untreated or treated for 24 h with 4 mm/l VAN. (**a**) Morphology. Cells were stained with Hoechst33342. Cellular and nuclear morphology was recorded by phase-contrast and fluorescence microscopy. Scale bar, 100 *μ*M. (**b**) Apoptosis was estimated as a percentage by counting the cells with typical apoptotic morphology. (**c**) Caspase activity. Cell lysate was collected for enzymatic assay of caspase activity. Data were expressed as means±S.D.; ^#^*P<0.05* *versus* Scramble group; *P<0.05 *versus* VAN group. Original magnification, × 200. Data are the representative of at least four separate experiments

Although VAN induced the expression of MBD2 *in vitro*, the role of MBD2 remains unclear in VAN-induced AKI. To verify its role, HK-2 cells were treated with MBD2 siRNA. As shown in Figure 2a, VAN markedly induced apoptosis in HK-2 cells, which was further inhibited by MBD2 siRNA. Similar result obtained from analysis of apoptosis rate and caspase activity (Figures 2b and c).

### MBD2 siRNA ameliorated VAN induced the expression of MBD2, BAX and active caspase 3

**Figure 3:**
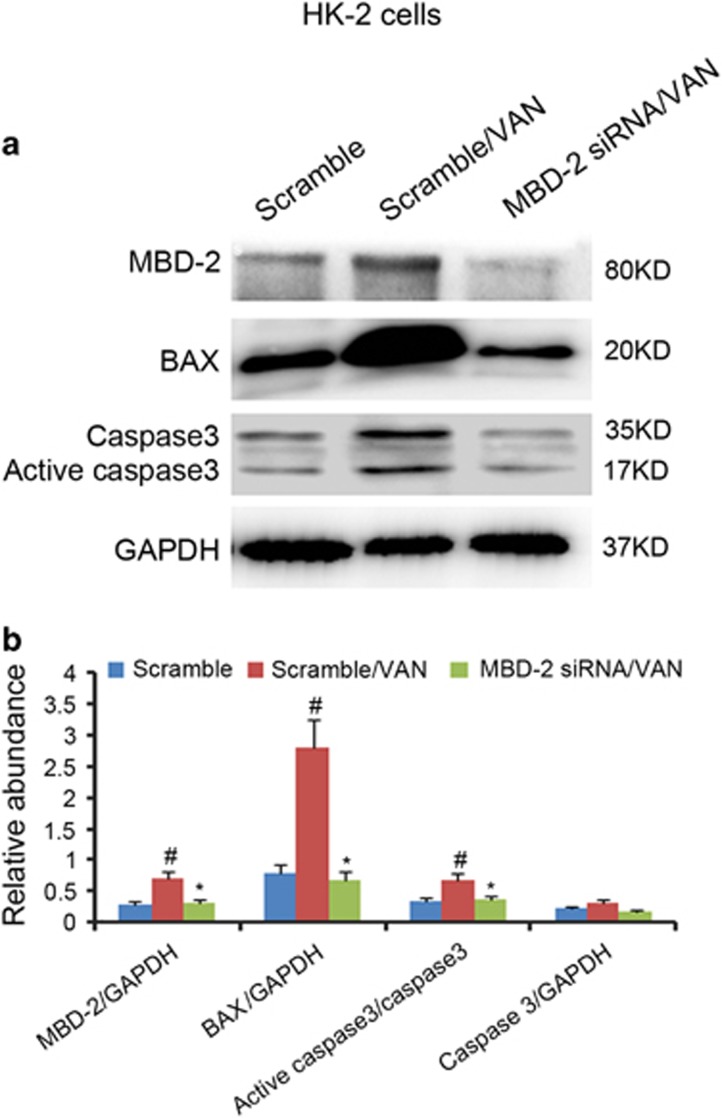
SiRNA MBD2 suppresses VAN induced the expression of p53 and its transcriptional target genes in HK-2 cells. HK-2 cells were transfected with 50 nmol/l MBD2 siRNA or scramble. Cells were then left untreated or treated for 24 h with 4 mm/l VAN. (**a**) The collect lysate was analyzed by immunoblot with p21, BAX, cleaved caspase 3, caspase 3 and GAPDH. (**b**) Immunoblot signals were quantified by densitometry, and normalized to internal actin control of GAPDH. Data were expressed as means±S.D.; ^#^*P*<0.05 *versus* Scramble group; *P<0.05 *versus* VAN group. Original magnification, × 200. Data are the representative of at least four separate experiments

Previous results have shown that VAN induced apoptosis-associated genes expression.^12, 15^ Therefore, we investigated whether inhibition of MBD2 reduced the expression of MBD2, BAX and active caspase 3. HK-2 cells were treated with MBD2 siRNA. As shown in Figure 3a, after VAN treatment, MBD2 siRNA showed lower expression of MBD2, BAX and less cleaved/active caspase 3, which was further verified by densitometric analysis of the immunoblots (Figure 3b). Together, these data further supported that MBD2 mediated VAN-induced HK-2 cells apoptosis.

### The expression of miR-301a-5p after VAN treatment is suppressed by MBD2 siRNA in HK-2 cells

**Figure 4:**
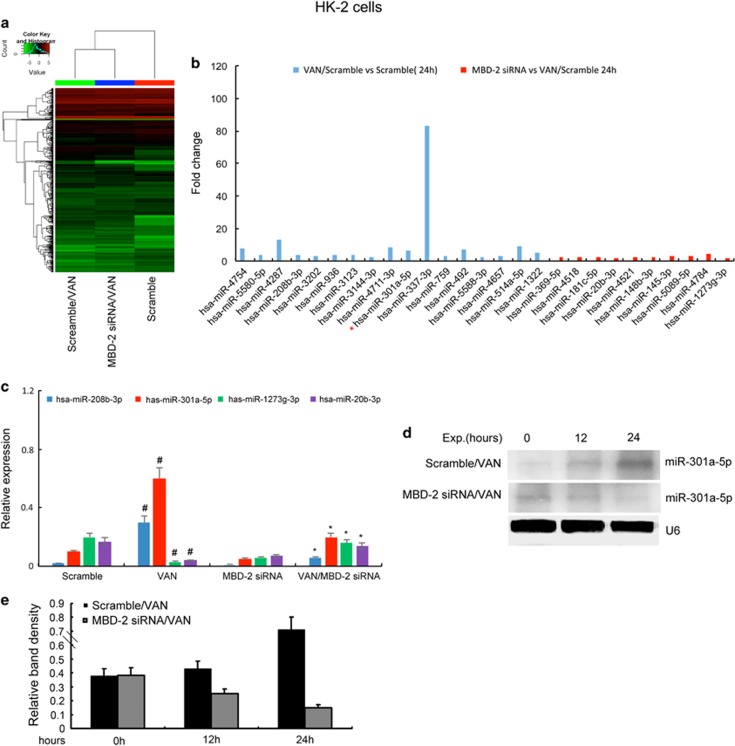
The induction of hsa-miR-301a-5p after VAN treatment is suppressed by siRNA MBD2 in HK-2 cells. HK-2 cells were transfected with 50 nmol/l MBD2 siRNA or scramble. Cells were then left untreated or treated for 24 h with 4 mm/l VAN. (**a**) Representative heat map of microRNA microarray analysis. The heat map was generated by the ΔCt values of all miRNAs. (**b**) The amount of each miRNA from the VAN group was divided by the amount of Scramble control to calculate the fold change. (**c**) Real-time PCR analysis of hsa-miR-208b-3p, hsa-miR-301a-5p, hsa-miR-1273g-3p and hsa-miR-20b-3p. The value of each miRNA is normalized by the signal of U6, an internal control. (**d**) Northern blot analyses of hsa-miR-301a-5p at 0–24h. Total RNA (10 *μ*g per lane) is analyzed by northern blotting as described in the Materials and methods section using a p32-labeled probe of hsa-miR-301a-5p. U6 is shown as an RNA loading control. (**e**) Northern blotting signals were quantified by densitometry, and normalized to internal U6 control. Data were expressed as means±S.D.; ^#^*P*<0.05 *versus* Scramble group; *P<0.05 *versus* VAN group. Data are the representative of at least four separate experiments

Although we have demonstrated that inhibition of MBD2 ameliorated HK-2 cells apoptosis, the mechanism remains unclear. We assumed that miRNAs might be a mechanistic link between the expression of MBD2 and the induction of apoptosis. A representative heat map of the microarray was shown in Figure 4a. Comparisons of miRNAs between different groups was shown in Supplementary Tables 1-3. Among the 1153 detectable miRNAs, 17 miRNAs were downregulated in MBD2 siRNA with VAN group, however, 10 miRNAs were upregulated in MBD2 siRNA with VAN group (Figure 4b). Of note, the two upregulated miRNAs, miR-1273g-3p suppressed apoptosis in HSCs,^28^ and miR-20b promoted apoptosis in P19 cell lines.^28^ The two downregulated miRNAs, miR-208b-3p induced apoptosis in myocardial cell,^29^ however, the role of miR-301a-5p in apoptosis remains unclear (Figure 4b). The expression of miR-208b-3p, miR-301a-5p, miR-1273g-3p and miR-20b-3p was confirmed by real-time PCR (Figure 4c), which is consistent with microarray analysis. As the most remarkable changes were seen in miR-301a-5p, the studies were extended and its expression was also assessed by northern blot analyses in HK-2 cells. After VAN treatment, the miR-301a-5p expression increases notably by 12 h, and by 24 h a steep increase was observed. The expression was markedly reduced with the MBD2 siRNA treatment (Figures 4d and e). These data demonstrate that MBD2 regulates the expression of miR-301a-5p.

### MBD2 activates methylated miR-301a-5p promoter activity by suppressing methylation of promoter

**Figure 5:**
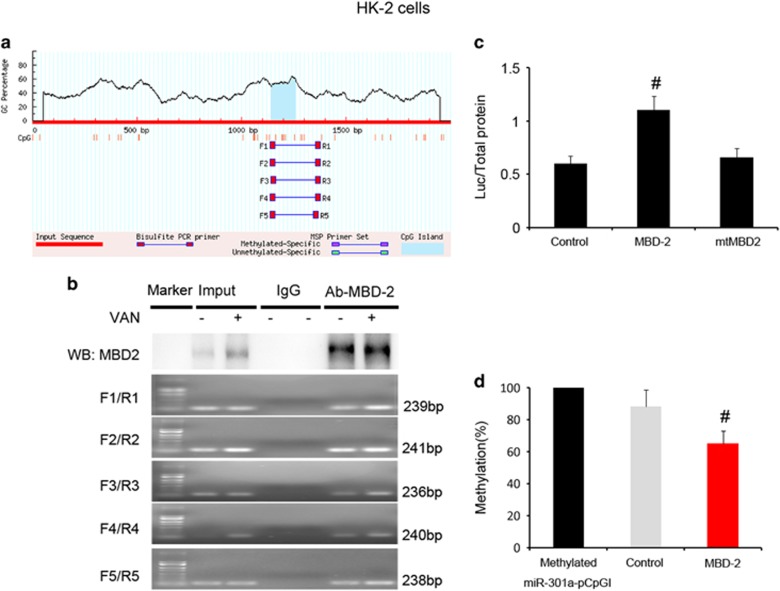
MBD2 directly binds to CpG island of miR-301a-5p promoter and activates it by suppressing methylation of promoter. (**a**) CpG island of miR-301a-5p promoter was predicted, and five primer pairs were designed by software of MethPrimer 2.0. (**b**) ChIP assays were performed with chromatin material isolated from HK-2 cells treated with VAN. Precipitates with MBD2 or without antibody (input) were used as template for PCR detection of the potential MBD2 binding sites1-5 (mBS1-5). (**c**) Relative luciferase activity in HK-2 cells. (**d**) CpG-DNA methylation of the miR-301a-5p promoter region. Data were expressed as means±S.D.; ^#^*P*<0.05 *versus* Control group. Data are the representative of at least four separate experiments

As MBD2 involved in methylation regulation, we guess that MBD2 could activate miR-301a-5p by suppression of methylation. First, the bioinformatics analysis using the MethPrimer, Promoter2.0 (http://www.urogene.org/cgi-bin/methprimer2/MethPrimer.cgi) (for CpG islands prediction and primer design), the miR-301a-5p promoter sequence showed the presence of one CpG islands (Figure 5a), and then carried out a chromatin immunoprecipitation (ChIP) assay to determine the interaction of MBD2 with miR-301a-5p promoter region in HK-2 cells. As shown in Figure 5b, the antibody directed against MBD2 immunoprecipitated the DNA fragments from HK-2 cells containing the potential binding sites of mBS1-5, supporting the hypothesis that MBD2 can physically interact with the miR-301a-5p promoter region. Furthermore, we construct CpG-free pCpGI luciferase reporter plasmid that included CG DNA methylation target sequences in promoter region of miR-301a-5p. After co-transfected with MBD2 plasmid, the transcriptional activity of methylated miR-301a-5p pCpGI is significantly increased, it is not increased with the mutant plasmid of MBD2 methylated DNA binding domain deletion (Figure 5c). We examined whether the binding of MBD2 with the ectopic miR-301a-5p promoter region leads to a change in its methylation state. Compared with naked methylated DNA, the methylated miR-301a-5p pCpGI was demethylated in endogenous MBD2-bound DNA, the demethylation of it was further enhanced in ecpotic MBD2 expression (Figure 5d). These data show that binding of MBD2 to promoter of miR-301a-5p is associated with demethylation.

### MiR-301a-5p promoted HK-2 cells apoptosis during VAN treatment

**Figure 6:**
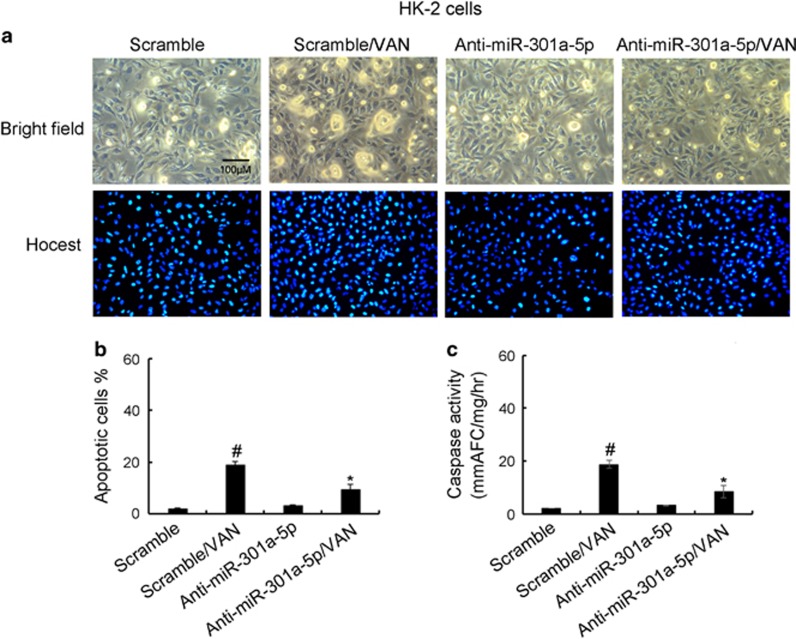
Inhibition of miR-301a-5p on VAN-induced apoptosis in HK-2 cells. HK-2 cells were transfected with 100 nmol/l LNA-modified antisense oligonucleotide of miR-301a-5p (anti-miR-301a-5p) or LNA-modified oligonucleotide of the scrambled sequence (scramble). Cells were then left untreated or treated for 24 h with 4 mm/l VAN. (**a**) Morphology. Cells were stained with Hoechst33342. Cellular and nuclear morphology was recorded by phase-contrast and fluorescence microscopy. Scale bar, 100 *μ*M. (**b**) Percentage of apoptotic cells was estimated by morphological methods. (**c**) Caspase activity. Cell lysate was collected for an enzymatic assay of caspase activity. Data were expressed as means±S.D.; ^#^*P*<0.05 *versus* Scramble group; *P<0.05 *versus* VAN group. Original magnification, × 200. Data are the representative of at least four separate experiments

Although MBD2 induced the expression of miR-301a-5p in HK-2 cells, the role of miR-301a-5p remains unclear in VAN-induced cells apoptosis. To clarify its role, HK-2 cells were treated with anti-miR-301a-5p treatment. As shown in Figure 6a, VAN markedly induced apoptosis in HK-2 cells, which was further inhibited by anti-miR-301a-5p. Similar result obtained from analysis of apoptosis rate and caspase activity (Figures 6b and c).

### MiR-301a-5p directly suppressed the expression of MITF, HDGF, and inhibited the MDM-4 expression to upregulation of p53 during VAN treatment in HK-2 cells

**Figure 7:**
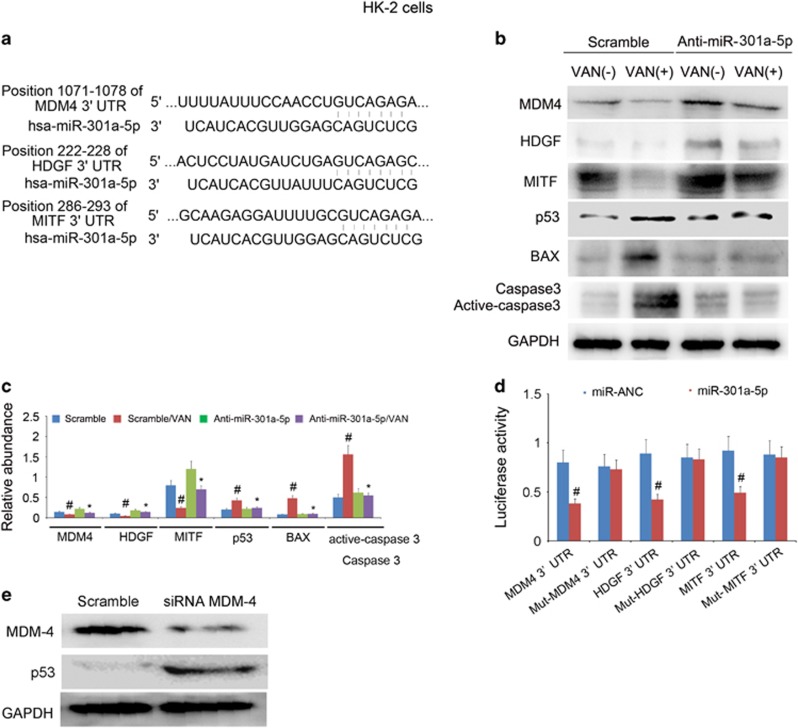
MiR-301a-5p promoted apoptosis by suppressing the expression of anti-apoptosis genes of HDGF and MITF, and also inhibiting MDM-4 for p53 expression in HK-2 cells. HK-2 cells were transfected with 100 nmol/l anti-miR-301a-5p or scramble. Cells were then left untreated or treated for 24 h with 4 mm/l VAN. (**a**) Putative miR-301a-5p complementary sequence in the 3′-UTR of human MDM-4, HDGF and MITF mRNA. (**b**) Immunoblot analysis of expression of MDM-4, HDGF, MITF, p53, BAX, cleaved caspase 3/caspase 3 and GAPDH. (**c**) Immunoblot signals were quantified by densitometry, and normalized to GAPDH of internal actin control. (**d**) Detected luciferase activity 24 h after cotransfection of miR-301a-5p analog (100 nM) or miR-ANC with 3’-UTR luciferase reporter vector of MDM-4, HDGF and MITF. (**e**) Relative protein levels of p53 24 h after the transfection of MDM-4 siRNA or Scramble. Data were expressed as means±S.D.; ^#^*P*<0.05 *versus* Scramble or MDM-4 3’-UTR group; *P<0.05 *versus* VAN group. Data are the representative of at least four separate experiments

Although miR-301a-5p is responsible for the HK-2 cells apoptosis, the regulation mechanism of it remains unclear. Both microphthalmia-associated transcription factor (MITF) and hepatoma-derived growth factor (HDGF) are considered as apoptosis suppressor genes.^30, 31, 32^ A large body of evidence indicates that p53 inactivation mainly dependent on upregulation of MDM-4 in tumor.^33^ In addition, we also identified MITF, HDGF and MDM-4 as target genes of miR-301a-5p by the prediction of miRBASE (http://mirdb.org/cgi-bin/target_detail.cgi?targetID=175447) (Figure 7a). Hence, we proposed that miR-301a-5p induced cell apoptosis by suppressing anti-apoptosis genes including HDGF and MITF, and also inhibiting the MDM-4 expression to upregulate p53. We found that VAN treatment significantly induced activation of p53 and the expression of BAX, less cleaved/active caspase 3, and reduced HDGF, MITF and MDM-4 protein, which was significantly reversed by anti-miR-301a-5p treatment (Figures 7b and c). To supply more direct evidence that miR-301a-5p targets HDGF, MITF, and MDM-4, wild-type and mutant luciferase reporter plasmids containing HDGF, MITF and MDM-4 3'-UTR region were co-transfected with a miR-301a-5p analog or a miRNA analog negative control (miR-NAC). Luciferase activity was markedly reduced at 24 h after the transfection in the presence of miR-301a-5p analog compared with miR-NAC (Figure 7d). In HK-2 cells, we also found that p53 was increased at 24 h after transfection with MDM-4 siRNA compared with negative control (Figure 7e). The results revealed that MBD2 induced miR-301a-5p to suppress the expression of HDGF, MITF, and inbitit MDM-4 for p53 activation.

### Deletion of MBD2 ameliorated renal dysfunction and renal injury in VAN nephrotoxic AKI mice

**Figure 8:**
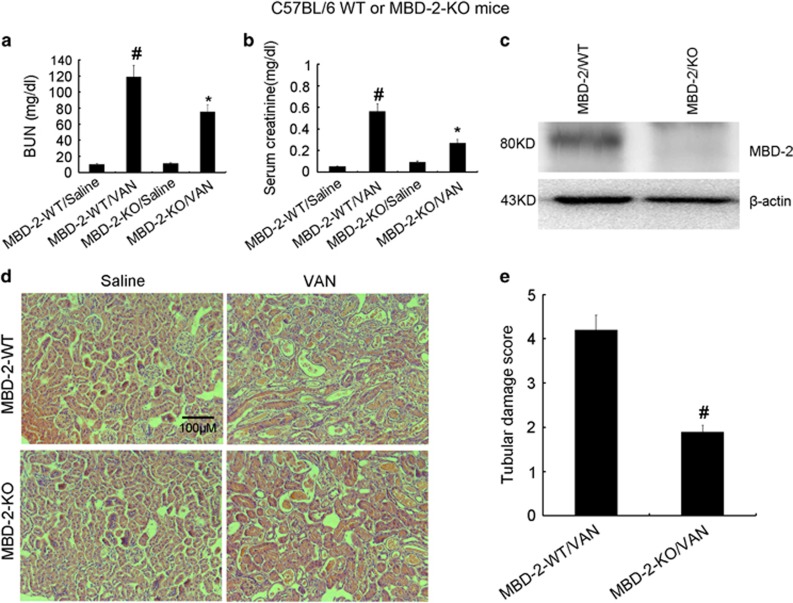
VAN-induced AKI is attenuated in MBD2-KO mice. Wild-type and MBD2-KO littermate mice were injected with 600 mg/kg VAN (n=8) or saline as control for 7 days. (**a** and **b**) Blood samples were collected for measurements of BUN and serum creatinine levels. (**c**) Kidneys were collected for immunoblot analysis of MBD2 and *β*-actin of an internal control. (**d**) Kidney cortical tissues were stained with hematoxylin–eosin to show histology (original magnification, × 200). (**e**) Tubular damage in VAN-treated cortical tissues was semiquantified as pathologic scores. Data were expressed as means±S.D.; ^#^*P*<0.05 *versus* Saline group; *P<0.05 *versus* VAN group. Data are the representative of at least four separate experiments

To assess the role of MBD2 in the pathogenesis of VAN nephrotoxic AKI, the wild-type and MBD2 KO littermate mice were treated with or without VAN. In the non-VAN treatment group, levels of BUN and serum creatinine were similarly low. At day 7, VAN treatment induced moderate renal failure in wild-type mice, which was significantly suppressed in MBD2 KO mice (Figures 8a and b). The immunoblot analysis demonstrated MBD2 was completely abolished in MBD2 KO mice compared with WT mice after VAN treatment (Figure 8c). Histologic analysis confirmed the VAN-induced kidney tissue damage as in MBD2-WT mice, which was significantly ameliorated in MBD2 KO mice (Figure 8d). In wild-type mice, the tubular damage score was 4.2 after VAN-induced AKI, whereas the score was markedly decreased to 1.8 after VAN-induced AKI for MBD2 KO tissues (Figure 8e).

### Deletion of MBD2 ameliorated apoptosis in VAN nephrotoxic AKI mice

**Figure 9:**
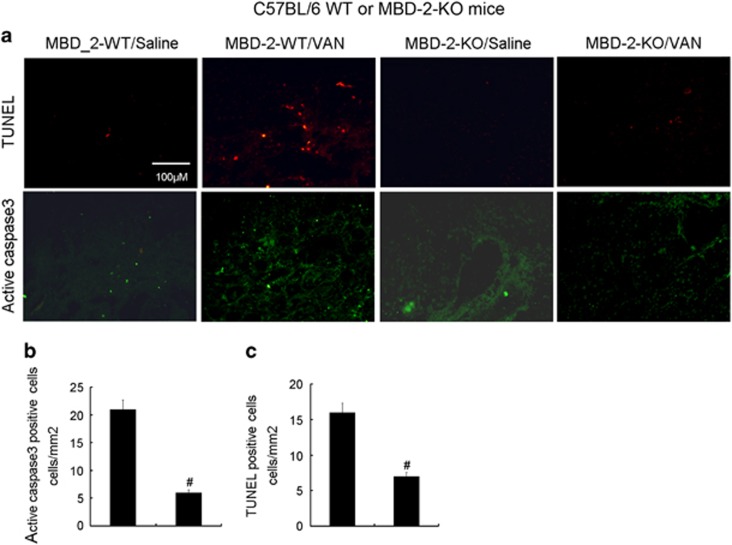
VAN-induced apoptosis is attenuated in MBD2-KO mice. Wild-type and MBD2-KO littermate mice were injected with 600 mg/kg VAN (*n*=8) or saline as control for 7 days. (**a**) Active caspase 3 and TUNEL assay to reveal apoptosis (original magnification, × 200). Both Active caspase 3 (**b**) and TUNEL-positive cells (**c**) were qualified in VAN-treated cortical tissues. Data were expressed as means±S.D.; ^#^*P*<0.05 *versus* Saline group; *P<0.05 *versus* VAN group. Data are the representative of at least four separate experiments

Previous results has shown that apoptosis has a pivotal role in the pathogenesis of AKI,^12, 34, 35, 36, 37^ whether MBD2 promotes apoptosis in VAN-induced AKI need to be investigated. Analysis of apoptosis in kidney cortical tissues using the terminal deoxynucleotidyl transferase mediated digoxigenin deoxyuridine nick-end labeling (TUNEL) staining and immunofluorescence of active caspase 3. The positive cells of TUNEL and active caspase 3 were lower in the kidney tissues of saline-injected mice, however, VAN induced significant increase of them in kidney cortical tissues in wild-type mice, which was markedly suppressed in MBD2-KO mice (Figure 9a). The number of positive cells of TUNEL and active caspase 3 in cortical and outer medulla regions further verified the above-mentioned observation (Figures 9b and c).

### The miR-301a-5p induced by VAN was blocked in MBD2-KO mice

**Figure 10:**
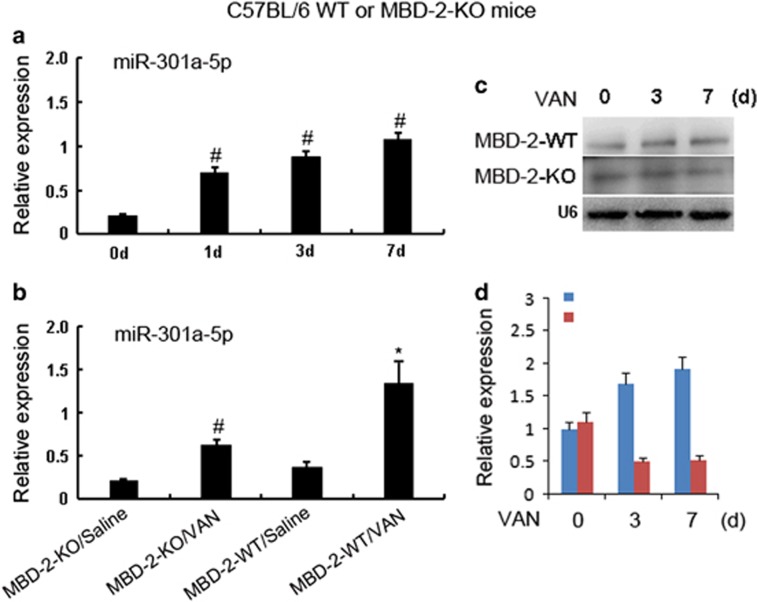
MiR-301a-5p expression is suppressed in p53 KO mice. Wild-type and MBD2-KO littermate mice were injected with 600 mg/kg VAN (*n*=8) or saline as control for 7 days. Whole-tissue lysate was analyzed for miR-301a-5p and U6 by real-time PCR or northern blot. (**a**) Real-time PCR showed the expression levels of miR-192-5p at indicated time points in VAN-induced AKI. (**b**) MiR-192-5p expression is reduced in MBD2 KO mice. (**c**) Representative northern blot is shown. (**d**) Northern blot signals were quantified by densitometry, and U6 is shown as an RNA loading control. Data were expressed as means±S.D.; ^#^*P*<0.05 *versus* Saline group; *P<0.05 *versus* VAN group. Data are the representative of at least four separate experiments

To further depth understanding of molecular mechanism of MBD2 for regulation of apoptosis, we focus on the miR-301a-5p. *In vitro* studies have demonstrated that miR-301a-5p mediates apoptosis during VAN treatment. In current experiment, first, real-time PCR showed that VAN induced significant increase of miR-301a-5p on day 1, and gradually increase of it on days 3 and 7 (Figure 10a). Second, as shown in the saline group (Figure 10b), level of miR-301a-5p was likewise low in these mice, after VAN treatment, it was markedly increased in MBD2-WT mice, which was ameliorated in tissues of MBD2-KO mice. Finally, northern blot analysis of the miR-301a-5p, on days 3 and 7 after VAN treatment, it was markedly downregulated in MBD2-KO mice than in WT mice (Figures 10c and d). These data confirmed our previous *in vitro* findings that miR-301a-5p is a target of MBD2.

### The target genes of miR-301a-5p were restored, and the expression of p53, BAX and active caspase 3 were suppressed during VAN treatment in MBD2-KO mice

**Figure 11:**
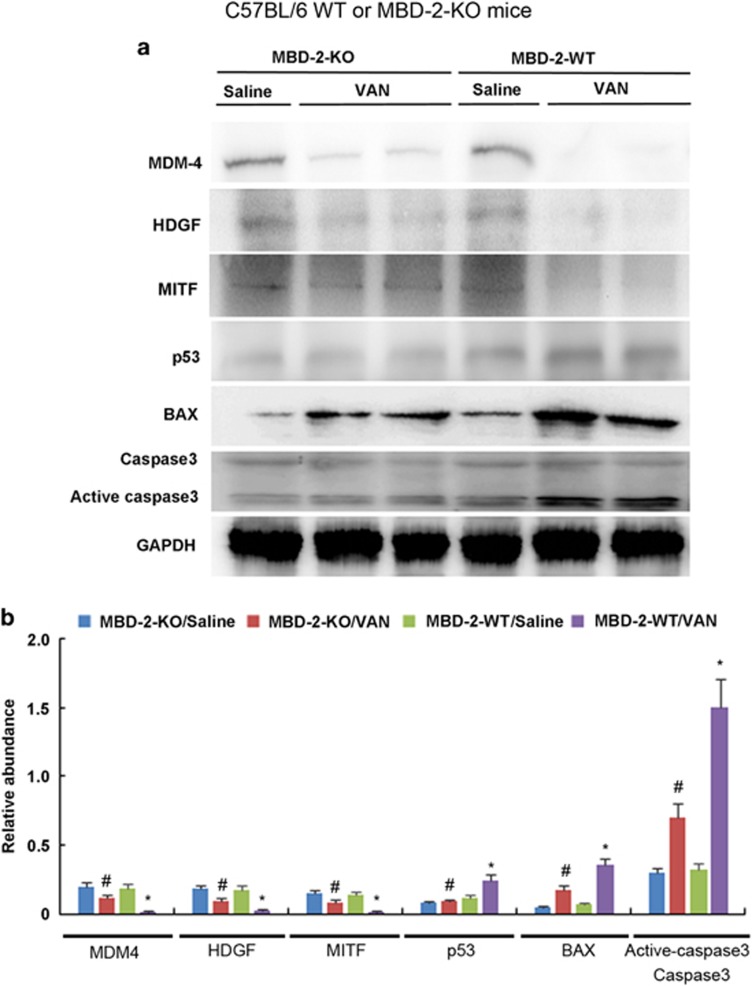
Suppressing of HDGF and MITF, and induction of p53 in VAN-induced AKI is reversed in MBD2 KO mice. Wild-type and MBD2-KO littermate mice were injected with 600 mg/kg VAN (n=8) or saline as control for 7 days. Whole-tissue lysate was analyzed for MDM-4, HDGF, MITF, p53, BAX, cleaved caspase 3/caspase 3 and GAPDH by using specific antibodies. (**a**) Representative immunoblot analysis. (**b**) Immunoblot signals were quantified by densitometry, and and normalized to GAPDH of internal actin control. Data were expressed as means±S.D.; ^#^*P*<0.05 *versus* Saline group; *P<0.05 *versus* VAN group. Data are the representative of at least four separate experiments

Our previous studies has demonstrated that cell apoptosis involved in VAN-induced AKI.^12, 15^In current study, we found that MBD2 induced MiR-301a-5p to suppress the expression of HDGF, MITF and MDM-4 for cell apoptosis during VAN treatment *in vitro*. To confirm the above observation, we analyzed the expression of these genes to determine their dependence on MBD2. As shown in Figure 11a, after VAN treatment, tissues of MBD2-KO mice showed higher HDGF, MITF, and MDM-4; lower p53, and BAX expression, and less cleaved/active caspase 3, which was further confirmed by densitometric analysis of immunoblots (Figure 11b). Collectively, these data for the first time demonstrate that MBD2 regulates key cell death regulatory genes during AKI.

### Deletion of MBD2 ameliorated inflammatory and the expression of MDM-4 in VAN nephrotoxic AKI mice

**Figure 12:**
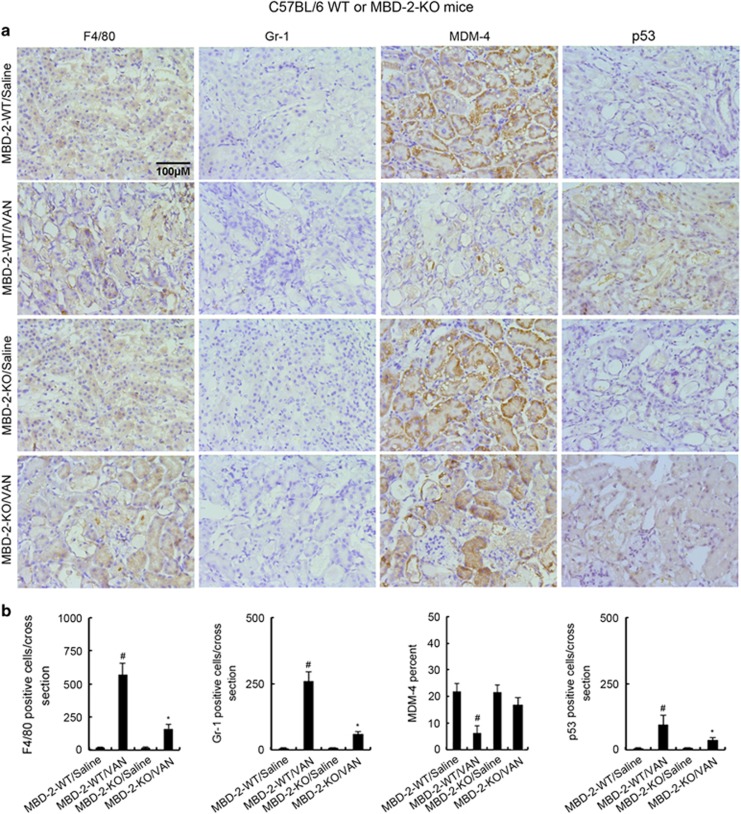
Infiltration of F4/80s and neutrophils, and expression of MDM-4 and p53 in VAN-induced AKI in MBD2 KO and wild-type mice. Wild-type and MBD2 KO littermate mice were injected with 600 mg/kg VAN (*n*=8) or saline as control for 7 days. (**a**) Immunohistochemical staining of macrophages, neutrophils and expression of MDM-4 and p53 (original magnification × 200). (**b**) Quantitation of positive staining cells per cross-sectional area. Data were expressed as means±S.D.; ^#^*P<0.05* versus Saline group; *P<0.05 *versus* VAN group. Data are the representative of at least four separate experiments

Recent studies demonstrated that inflammation also has an important role in VAN-induced AKI.^5, 6^ As MBD2 regulates expression of p53, latter involved in inflammation regulation, we proposed that inflammation was regulated by MBD2. To verify this, we examined the infiltration of inflammatory cells, and the expression of MDM-4 in VAN nephrotoxic AKI. As shown in Figures 12a and b, VAN induced the infiltration of neutrophils and macrophages into wild-type kidney tissues, and the low expression of MDM-4, whereas they were reversed in MBD2-KO tissues. The data verify that MBD2 also regulates inflammation during AKI.

### Inhibition of miR-301a-5p ameliorated renal dysfunction, renal injury in VAN nephrotoxic AKI mice

**Figure 13:**
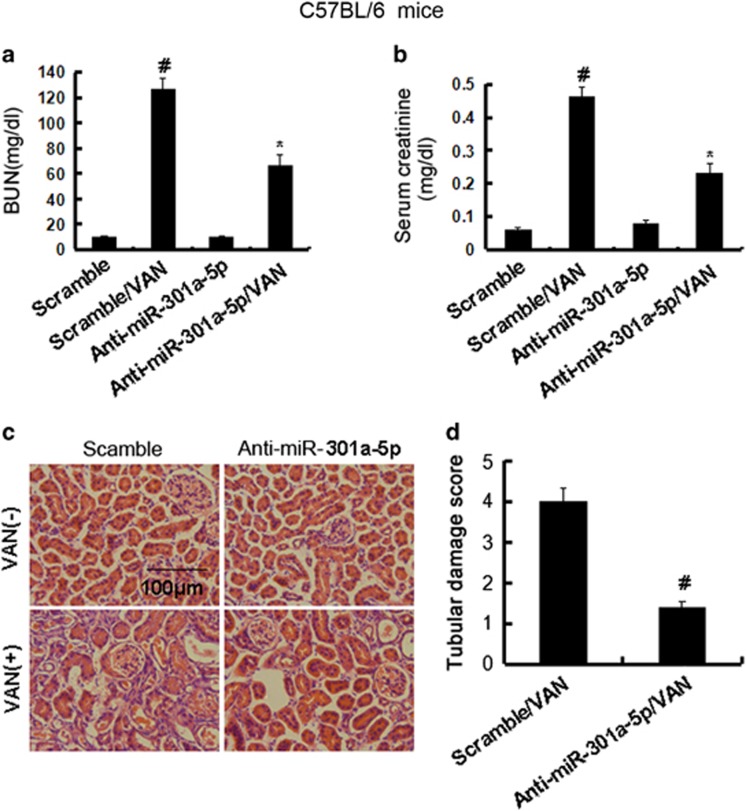
Blockade of miR-301a-5p reduced VAN-induced AKI. Male C57BL/6 mice were (**a**-**d**) injected with 600 mg/kg VAN (n=8) with or without 20 mg/kg LNA-modified antisense oligonucleotide of miR-301a-5p (anti-miR-301a-5p) or LNA-modified oligonucleotide of the scrambled sequence (scramble) for 7 days of examination. In **a** and **b**, blood samples were collected to measure BUN and serum creatinine (**a** and **b**). (**c**) Kidney cortical tissues were stained with hematoxylin-eosin to show histology (original magnification, × 200). (**d**) Tubular damage in VAN-treated cortical tissues was semiquantified as pathologic scores. Data were expressed as means±S.D.; ^#^*P<*0.05 *versus* Scramble group; *P<0.05 *versus* VAN group. Data are the representative of at least four separate experiments

Although inhibition of miR-301a-5p reduced the apoptosis *in vitro*, the role of miR-301a-5p in the pathogenesis of VAN nephrotoxic AKI remains unclear. Male C57BL/6 mice were injected with LNA-modified antisense oligonucleotide of miR-301a-5p (anti-miR-301a-5p) or LNA-modified oligonucleotide of the scrambled sequence (scramble), levels of BUN and serum creatinine were similarly low in these mice, indicating normal renal function. At day 7 of VAN treatment, scrambled mice developed moderate renal failure, with 121.2 mg/dl BUN and 0.48 mg/dl serum creatinine, whereas anti-miR-301a-5p mice had 62.6 mg/dl BUN and 0.24 mg/dl serum creatinine (Figures 13a and b). Histological analysis confirmed the VAN-induced kidney tissue damage, which was significantly ameliorated in anti-miR-301a-5p mice (Figure 13c). In scrambled mice, the tubular damage score was 3.9 after VAN AKI, whereas the score was markedly decreased to 1.3 after VAN AKI for anti-miR-301a-5p tissues (Figure 13d).

## Discussion

Although previous results reported that DNA methylation has a pivotal role in ischemic-induced AKI,^19, 20, 21^ how DNA methylation affects the development of AKI remains poorly understood. In present report, we focused on the MBD2, an epigenetic interpreter, to investigate the role and mechanism of VAN-induced AKI. Our study for the first time reported that global MBD2-KO significantly ameliorated VAN-induced AKI. Moreover, we have identified that MBD2 may induce miR-301a-5p to suppress anti-apoptosis genes including HDGF and MITF, and inhibit MDM-4 for p53 activation, together resulting in cell apoptosis and renal injury.

Our result for the first time showed that MBD2 was expressed in HK-2 cells and nuclei of normal kidney tissues, after VAN treatment, it was markedly increased at different time points *in vitro and vivo*, and mainly induced in the nuclei of widespread injury tubular cells *in vivo* (Figure 1). As we know, apoptosis had a pivotal role in VAN-induced AKI.^12, 35^ Although recent study has demonstrated that inhibition of MBD2 suppressed H_2_O_2_ induced endothelial cells apoptosis through activating ERK1/2 to phosphorylate BCL-2,^22^ the role and mechanism of VAN-induced HK-2 cell apoptosis remains unclear. Our current study demonstrated that inhibition of MBD2 using siRNA MBD2 and MBD2 KO also significantly ameliorated renal cell apoptosis (Figures 2 and 9). Interestingly, inflammation induced by VAN was also significantly suppressed in MBD-KO mice.

To further investigate the mechanisms of MBD2 on the activation of apoptosis, microarray analyses were carried out using HK-2 cells. The expression levels of miR-1273g-3p and miR-20b were found to be consistently low in VAN group, and they were increased in siRNA MBD2 with VAN group. Previous results have shown that miR-1273g-3p was associated with suppression of apoptosis in HSCs,^27^ whereas miR-20b was related with induction of apoptosis in P19 cell lines.^28^ The expression levels of miR-208b-3p and miR-301a-5p were consistently high in VAN group, which was decreased in siRNA MBD2 with VAN group. A recent study reported that miR-208b-3p might promote apoptosis in myocardial cell.^29^ Although numerous reports have now shown the involvement of miR-301a in various tumor types,^38, 39, 40, 41^ the regulation mechanism and role of miR-301a-5p in VAN-induced apoptosis need to be investigated. Our results indicated that MBD2 directly regulated miR-301a-5p, which was supported by evidences as described below. First, we found that miR-301a-5p was significantly upregulated by VAN treatment *in* *vitro and in vivo* at indicated time points, which was markedly suppressed in siRNA MBD2 and MBD2 KO mice (Figures 4d and e, and Figure 10). Second, using ChIP assays, we demonstrated that MBD2 could physically interact with the promoter region of miR-301a-5p (Figures 5a and b). In addition, MBD2 activates the miR-301a-5p promoter via reducing the metalyation (Figures 5c and d).

**Figure 14:**
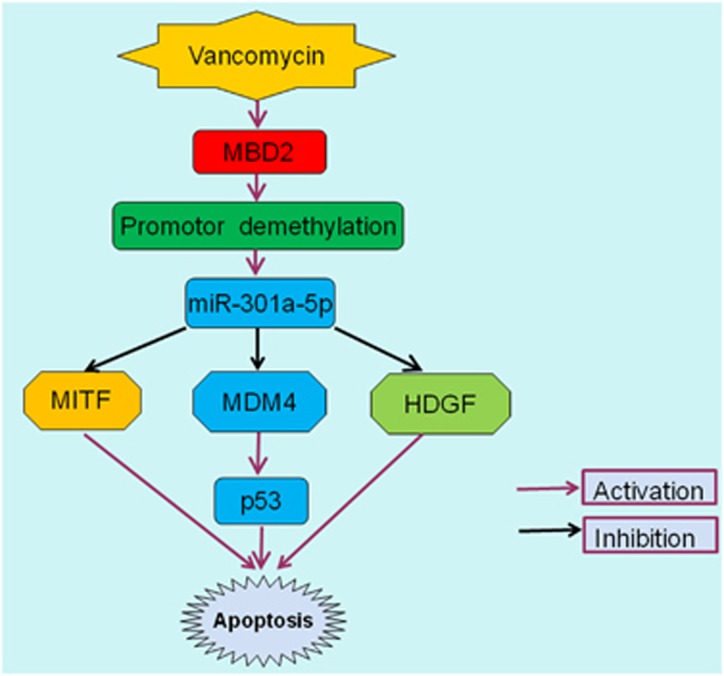
The role and molecular mechanism of MBD2 in VAN-induced AKI. After VAN treatment, MBD2 increases its expression by suppression of promoter methylation of miR-301a-5p. Subsequently, upregulation of miR-301a-5p induced cell apoptosis via directly suppressing the expression of HDGF and MITF, and also inhibiting MDM-4 to increase expression of p53

These data for the first time suggest that inhibition of miR-301a-5p significantly ameliorates VAN-induced renal cell apoptosis and renal injury *in vitro and in vivo* (Figures 7 and 13), which indicated that miR-301a-5p may act as an apoptosis promoter, and thus may be referred as a potential therapeutic target for VAN-induced AKI. However, miR-301a-5p as a potential new therapeutic target for other diseases could be further explored. Mechanically, miR-301a-5p suppressed anti-apoptosis genes including HDGF and MITF, and also inhibited MDM-4 for p53 activation. First, inhibition of miR-301a-5p markedly increased expression levels of anti-apoptosis genes of HDGF and MITF, and also upregulated MDM-4 to inhibit p53 expression (Figures 6a and b). Second, our luciferase reporter assay identified MITF, HDGF and MDM-4 as a target gene of miR-301a-5p in HK-2 cells, and further MDM-4-silencing in HK-2 cells significantly enhanced the p53 accumulation (Figures 6c and d), which was supported by a previous study that MDM-4 was an essential for negative regulation of p53.^33^ Our previous result has shown p53 mediated VAN and ischemic induced renal cell apoptosis and inflammation.^12, 37, 38^ Surprisingly, using MBD2 KO, VAN suppressed the expression of anti-apoptosis genes of MITF and HDGF was reversed, moreover, VAN-induced p53 expression were significantly downregulated via upregulation of MDM-4 *in vivo* (Figure 11), which might together explain the attenuation of apoptosis and inflammation. Collectively, these data reveal a novel regulatory mechanism by which MBD2 induces miR-301a-5p to suppress the expression of HDGF and MITF, and also inhibit MDM-4 for p53 activation in HK-2 cells (Figure 14). This mechanism seems to work not only in HK-2 cells, but also in mice.

In conclusion, we for the first time demonstrated that MBD2 had a pivotal role in renal injury, which was supported by assuagement of VAN-induced renal cell apoptosis using siRNA MBD2 and global knockout MBD2 mice. In HK-2 cells and mouse models, inhibition of MBD2 downregulates miR-301a-5p to restore anti-apoptosis genes expression including HDGF and MITF, and also increase MDM-4 expression for reduction of p53. Our present study suggests that MBD2 may be a therapeutic target of VAN-induced AKI; however, it could be studied further as a potential target for other type AKI in the future work.

## Materials and methods

### Reagents and antibodies

Antibodies were purchased from the following sources: polyclonal anti-MBD2, MDM-4, p53, Gr-1, F4/80 and active caspase 3 from Cell Signaling Technology (Beverly, MA, USA); polyclonal anti-p21 and anti-BAX (N-20) from Santa Cruz Biotechnology (Dallas, TX, USA). All secondary antibodies were from Thermo Fisher Scientific (Waltham, MA, USA). Carbobenzoxy-Asp-Glu-Val-Asp-7-amino-4-trifluoromethyl coumarin (DEVD- AFC), and 7-amino-4-trifluoromethyl coumarin (AFC) were purchased from Enzyme Systems Products (Livermore, CA, USA). VAN was obtained from Sigma-Aldrich (St. Louis, MO, USA). *In* *situ* cell death detection kit was obtained from Roche Applied Science (Indianapolis, IN, USA). Construction of methylation promoter of miR-301a-5p CpG-free pCpGI luciferase reporter, MBD2 and mtMBD2 (the deletion of the methylated DNA-binding domain) plasmids as described previously.^42, 43^

### Cell culture and treatments

HK-2 cells were cultured in Dulbecco’s modified Eagle’s medium (Sigma-Aldrich) supplemented with 10% fetal bovine serum, 0.5% penicillin and streptomycin in 5% CO_2_ incubator at 37° C. For transfection experiment, transfection of miR-301a-5p analog (100 nM) or negative control (miR-neg; Sigma, St. Louis, MO, USA) were used, and then HK-2 cells were treated with or without VAN (4 mm/l) for 24 h.

### ChIP analysis

ChIP assays were performed using a ChIP kit (Millipore, Boston, MA, USA) with primary antibodies against MBD2.^35, 44, 45^ Precipitated DNAs were detected by PCR using specific primers: mBS1: 5′-GATTATTTTGGTTAAGAGGGTGAAAT-3′ and 5′-ACAACTTTAAAAATACCCCAAAACA-3′, mBS2: 5′-GAGATTATTTTGGTTAAGAGGGTGA-3′, and 5′-ACAACTTTAAAAATACCCCAAA ACA-3′, mBS3: 5′-TATTTTGGTTAAGAGGGTGAAATTT-3′ and 5′-ACAACTTTAAAAATACCCCAAAACA-3′, mBS4: 5′-GATTATTTTGGTTAAGAGGGTGAAAT-3′ and 5′-TACAACTTTAAAAATACCCCAAAACA-3′, mBS5: 5′-GATTATTTTGGTTAAGAGGGTGAAAT-3′ and 5′-AAAAATACCCCAAAACAAAAAACTT-3′.

### Methylated CpG-DNA immunoprecipitation

The methylated CpG-DNA immunoprecipitation assay was carried out according to the manufacturer’s instructions (Zymo Research, Orange County, CA, USA) as previously described.^46^ Briefly, purified genomic DNA was sheared to fragments of 200 bp using DNA Shearase (Zymo Research), which were used for methylated CpG immunoprecipitation. Methylated DNA was recovered and subjected to PCR analysis on an ABI Onestepplus Real-Time PCR system (Shanghai, China).

### Animal model

MBD2 global knockout mice were purchased from Cyagen Biosciences Co., Ltd (Guangzhou, People’s Republic of China). The mice were intraperitoneally injected with a single dose of VAN at 600 mg/kg. In addition, the experiment on role of miR-301a-5p, male C57BL/6 mice were injected by tail vein with or without 20 mg/kg LNA-modified antisense oligonucleotide of miR-301a-5p (anti-miR-301a-5p) or LNA-modified oligonucleotide of scrambled sequence (scrambled) for 7 days. The control group was administered with saline. Animal experiments were performed in accordance with a protocol approved by the Institutional Committee for the Care and Use of Laboratory Animals of Second Xiangya Hospital, China. Mice were killed on day 7 after VAN or saline. The kidneys were harvested for various morphological and biochemical studies.

### Renal function, histology and TUNEL assay

BUN and serum creatinine were measured with commercial kits from Stanbio Laboratory (Boerne, TX, USA).^12, 36, 37^ For the histological analysis, kidney tissues fixed with 4% buffered paraformaldehyde were embedded in paraffin, and 4* μ*m thick sections were prepared. Hematoxylin–eosin staining was used for the sections, followed by a blind examination. The score of tissue damage was assessed according to the percentage of damaged tubules: 0, no damage; 1, <25% damage; 2, 25–50% damage; 3, 50–75% damage; 4, >75% damage. The criteria of tubular damage includes the loss of tubular dilation, brush border, tcast formation and cell lysis. The *In Situ* Cell Death Detection Kit from Roche Applied Science was using for TUNEL assay. For quantification, we randomly selected 10–20 fields from each tissue section to count the TUNEL-positive cells per millimeter.

### Immunohistochemistry, immunofluorescence and Immunoblot analysis

Immunohistochemical or immunofluorescence analyses were performed by using anti-MBD2, p53, MDM-4, Gr-1, F4/80 or active caspase 3 according to the previous protocol.^12, 47, 48^ Total numbers of positive cells for MBD2 and p53 (as identified by nuclear staining) was quantified by counting the number of stained cells per field. We collected 25–30 images of a kidney from each animal at × 20 magnification. Immunoblot was carried out as previously described.^12^ Briefly, cells or kidney tissues were treated with a lysis buffer (Sigma) containing phosphatase inhibitors (Calbiochem, Germany). Each well was loaded with equal amounts of proteins for electrophoresis using SDS–PAGE gel, followed by transferring to polyvinylidene fluoride membranes. Primary antibodies were incubated with membranes overnight at 4 °C, and probed by the horseradish peroxidase-conjugated secondary antibodies. Bands of target and internal control protein were separately outlined, and then gray level was analyzed using an Image J software (NIH, Bethesda, USA), the gray ratio of target protein *versus* internal control protein was calculated.

### Real-time PCR analysis of miRNAs

Total RNA of kidney cortical tissues or HK-2 cells was extracted by the mirVanamiRNA isolation kit (Applied Biosystems/Ambion, Austin, TX, USA) according to the manufacturer's instruction. Using miRNAqRT-PCR Detection Kit (Ambion), 40 ng of total RNA was reverse-transcribed to cDNA. Real-Time PCR was performed using the Taqman miRNA assay kit (Applied Biosystems) including the sequence-specific primers for cDNA synthesis and Taqman probes for real-time PCR. Quantification was done using ΔCt values.

### Northern blot analysis of miRNAs

The mirVanamiRNA isolation kit was used to extract total RNA. A denaturing 10% polyacrylamide gel was used to run 10 *μ*g of RNA, and then transferred onto the Hybond-N+ membrane (Amersham, Piscataway, NJ, USA), subjected to UV light irradiation for 4 min and baked at 80°C for 1 h. Subsequently, the membrane was pre-hybridized with ULTRA hyb-Oligo Hybridization Buffer (Applied Biosystems/Ambion) for 1 h, and then subjected to hybridization with ^32^p-labeled antisense specific miRNA probe overnight at 37 °C. Then the membrane was washed in 2 × SSC buffer (0.1% SDS) and exposed to X-ray film at −80 °C.

### Statistical analysis

Data were expressed as mean±S.E.M. One-way ANOVA followed by the Tukey’s post-hoc test was used to compare multiple treatment groups. Two-way ANOVA was used to assess the statistical significance of the differences between multiple treatment groups at different time points. Statistical significance was set at *P*<0.05.

## Publisher’s Note

Springer Nature remains neutral with regard to jurisdictional claims in published maps and institutional affiliations.
