# Supplementary material for: MBD2 upregulates miR-301a-5p to induce kidney cell apoptosis during vancomycin-induced AKI
Source: Cell Death Dis. 2017 Oct 12;8(10):e3120–. doi: 10.1038/cddis.2017.509 (PMC5682674; doi:10.1038/cddis.2017.509)
Supplement: Supplementary Table 1 [file cddis2017509x1.pdf]

Condition pairs: NV24h vs 24NC
Fold Change cut-off: 2.0

Column "ID": array ID of the probes, each miRNA always has its unique probe, but some miRNAs may have two different probes.
Column "Name": the name of each miRNA.
Column "Fold change": the ratio of normalized intensities between two conditions (use normalized data, ratio scale).
Column "ForeGround": the foreground intensity of each probe.
Column "ForeGround-BackGround": the signal of the probe after background correction.
Column "Normalized": the normalized ratio of the microRNA. Median Normalization Method was adopted.

VAN vs control 2.0 fold up regulated miRNAs							
		Fold chang	ForeGround		eGround-BackGro		Normalize
ID	Name	VAN vs control	control	VAN	control	VAN	control
17928	hsa-miR-181a-2-3p	5.10886	66.5	128.5	13	58.5	0.05634
42795	kshv-miR-K12-3-5p	3.45751	62.5	109	11	33.5	0.04767
146117	hsv1-miR-H6-3p	11.9837	56.5	117.5	4.5	47.5	0.0195
145678	hsa-miR-150-5p	2.49321	77	131	25.5	56	0.11051
168640	hsa-miR-4475	5.29481	115	339.5	58	270.5	0.25135
168861	hsa-miR-4754	7.83358	58.5	100.5	5	34.5	0.02167
169170	hsa-miR-4472	4.78022	64	106	9.5	40	0.04117
147722	hsa-miR-4306	2.2059	148.5	254.5	96.5	187.5	0.4182
147595	hsa-miR-3178	3.15296	149.5	335	96.5	268	0.4182
27565	hsa-miR-423-5p	3.92539	215.5	639	165	570.5	0.71506
168954	hsa-miR-5580-5p	3.74649	69.5	133	20	66	0.08667
147767	hsa-miR-4279	3.382	109	214.5	47.5	141.5	0.20585
148682	hsa-miR-483-3p	3.77936	130.5	325.5	76	253	0.32936
169308	hsa-miR-4503	2.20573	79	108	17.5	34	0.07584
169282	hsa-miR-4290	3.5406	124.5	253.5	59	184	0.25569
169034	hsa-miR-642b-5p	3.52108	91	176	34.5	107	0.14951
27568	hsa-miR-744-5p	3.28709	137	317	86	249	0.3727
42832	hsa-miR-638	3.82218	61.5	122.5	15	50.5	0.06501
146090	hsv1-miR-H7-3p	3.96637	168.5	479.5	118.5	414	0.51354
147938	hsa-miR-4287	12.8667	66	188.5	10.5	119	0.0455
148687	hsa-miR-1908-5p	3.19424	111.5	237.5	59	166	0.25569
148234	hsa-miR-3667-5p	2.00347	81	113	25.5	45	0.11051
148284	hsa-miR-208b-3p	3.62238	167	446.5	118	376.5	0.51138
169320	hsa-miR-4468	3.22615	117.5	237	60	170.5	0.26002
169319	hsa-miR-3136-3p	2.01683	91.5	144	42.5	75.5	0.18418
42502	hsa-miR-204-3p	6.00989	114	404.5	63	333.5	0.27302
145633	hsa-let-7d-3p	3.51058	120	287.5	70.5	218	0.30553
168919	hsa-miR-4456	2.95645	248	578.5	194.5	506.5	0.8429
168776	hsa-miR-4795-3p	5.90777	105	355	54	281	0.23402
168998	hsa-miR-4508	9.71313	55	112	4.5	38.5	0.0195
42782	hcmv-miR-UL148D	2.90637	69	102	12.5	32	0.05417
46336	hsa-miR-1284	7.47894	107	393	48.5	319.5	0.21018
42581	hsa-miR-513a-5p	7.23425	76.5	205	21.5	137	0.09317

146158	hsa-miR-3202	3.36252	128.5	303	78.5	232.5	0.3402
169050	hsa-miR-4787-5p	4.20245	189.5	534	124	459	0.53738
27537	ebv-miR-BART13-3p	2.48347	83.5	124	24	52.5	0.10401
168727	hsa-miR-4426	4.37091	61.5	107	10	38.5	0.04334
168971	hsa-miR-4449	9.79197	63.5	174.5	12	103.5	0.052
169087	hsa-miR-149-3p	5.34012	63.5	132	13.5	63.5	0.0585
169138	hsa-miR-4504	2.19554	116.5	192	60.5	117	0.26219
42906	ebv-miR-BHRF1-1	3.15078	97	196	44.5	123.5	0.19285
146196	hsa-miR-711	7.77334	104	409.5	49	335.5	0.21235
148085	hsa-miR-3687	11.5044	61	150	7.5	76	0.0325
11037	hsa-miR-299-3p	2.67931	78.5	132.5	25	59	0.10834
168765	hsa-miR-4448	4.05465	65	109	10.5	37.5	0.0455
168928	hsa-miR-4431	2.40121	110	191.5	56.5	119.5	0.24485
169079	hsa-miR-4667-5p	3.61063	118.5	264.5	61	194	0.26436
169082	hsa-miR-1275	2.28921	147	256	91.5	184.5	0.39653
169399	hsa-miR-4750-5p	3.5836	113	254.5	57.5	181.5	0.24919
145768	hsa-miR-665	4.96098	112	331.5	59.5	260	0.25785
147604	hsa-miR-4285	5.44945	80.5	195.5	25	120	0.10834
147804	hsv1-miR-H17	13.7047	64	244	14	169	0.06067
148263	hsa-miR-1273e	5.72381	64.5	136	12	60.5	0.052
148216	hsa-miR-3907	2.09515	168.5	281.5	110	203	0.47671
168893	hsa-miR-4505	4.26717	67	128.5	14.5	54.5	0.06284
169179	hsa-miR-4650-5p	4.85439	88.5	203	29	124	0.12568
42929	hsa-miR-25-5p	3.22055	78	140.5	24.5	69.5	0.10618
42869	hsa-miR-936	4.00059	64	107	10.5	37	0.0455
46258	hsa-miR-1184	3.54079	103.5	229.5	50.5	157.5	0.21885
148032	hsa-miR-3685	3.91759	88.5	189.5	35.5	122.5	0.15385
17488	kshv-miR-K12-6-3p	5.52842	81.5	185	23	112	0.09967
169285	hsa-miR-4467	3.18477	308	773	249	698.5	1.07909
169379	hsa-miR-4694-3p	7.00103	62	141.5	12	74	0.052
169232	hsa-miR-3156-3p	2.85188	114	226	62.5	157	0.27086
169395	hsa-miR-4484	2.11018	106.5	153	46	85.5	0.19935
146072	hsa-miR-1469	4.16277	83	173	24	88	0.10401
147942	hsa-miR-4268	4.46552	86.5	194	30	118	0.13001
148622	hsa-miR-877-3p	2.60699	70.5	112.5	13.5	31	0.0585
169239	hsa-miR-4732-5p	2.02978	89.5	163.5	33	59	0.14301
42514	hsa-miR-937-3p	2.01393	345	590	287.5	510	1.24594
147651	hsa-miR-3123	3.06773	84	142.5	23.5	63.5	0.10184
169375	hsa-miR-660-3p	4.98101	107.5	322	55.5	243.5	0.24052
169380	hsa-miR-3124-3p	2.22203	149.5	259.5	93.5	183	0.4052
169189	hsa-miR-4795-5p	4.87233	65	133	12	51.5	0.052
147923	hsa-miR-3142	2.59497	121	212.5	63	144	0.27302
147631	hsa-miR-4258	6.43337	60.5	101.5	6	34	0.026
147930	hsa-miR-3144-3p	2.57808	76	123.5	24	54.5	0.10401
148156	hsa-miR-3686	2.38306	158.5	292	106	222.5	0.45937
168941	hsa-miR-4501	3.66324	91	191.5	37.5	121	0.16251
169116	hsa-miR-4788	7.35843	65.5	158.5	13.5	87.5	0.0585
169012	hsa-miR-4711-3p	8.62829	65	107.5	5	38	0.02167
42702	hsa-miR-30c-1-3p	7.7003	78	227.5	23	156	0.09967
168638	hsa-miR-4530	5.4116	88	224.5	30	143	0.13001
169024	hsa-miR-3960	2.16656	371	674	311	593.5	1.34778

169028	hsa-miR-4708-3p	3.14939	177	400	119.5	331.5	0.51788
13137	hsa-miR-518e-5p/hs	8.20513	73	162.5	11	79.5	0.04767
42458	hcmv-miR-US25-1-3	11.0692	70.5	145.5	6	58.5	0.026
148678	hsa-miR-301a-5p	6.54346	87	236.5	27.5	158.5	0.11918
147738	hsv2-miR-H20	3.71553	93.5	190.5	33	108	0.14301
168935	hsa-miR-4687-3p	6.06677	73	158.5	16	85.5	0.06934
168672	hsa-miR-1587	9.1581	66.5	134.5	7.5	60.5	0.0325
17492	sv40-miR-S1-5p	3.35957	126	290	73.5	217.5	0.31853
42673	hsa-miR-337-3p	82.877	76.5	1858.5	24.5	1788.5	0.10618
17668	hsa-miR-552-3p	6.96662	65	139.5	11	67.5	0.04767
23767	hsa-miR-759	2.90637	67	103.5	12.5	32	0.05417
147790	hsv2-miR-H7-3p	7.77892	80.5	256	27	185	0.11701
168978	hsa-miR-371b-5p	3.42407	178	449	125	377	0.54171
169228	hsa-miR-4698	4.10601	82.5	179	30	108.5	0.13001
169110	hsa-miR-4497	4.71885	330.5	1225	278	1155.5	1.20477
42661	hsa-miR-492	6.91502	63.5	103.5	5.5	33.5	0.02384
168841	hsa-miR-5588-3p	2.38211	114.5	187	56	117.5	0.24269
46731	hsa-miR-4657	2.72715	197	405	140.5	337.5	0.60888
169212	hsa-miR-514a-5p	9.36624	63.5	122.5	6	49.5	0.026
169313	hsa-miR-4800-3p	5.12317	293.5	1150.5	238	1074	1.03142
169388	hsa-miR-663a	12.9141	66	157	8	91	0.03467
46408	hsa-miR-1322	42.8009	64	268.5	5	188.5	0.02167
168944	hsa-miR-4707-5p	4.66284	71.5	133.5	14	57.5	0.06067
168660	hsa-miR-5000-5p	17.8552	61.5	166.5	5.5	86.5	0.02384

VAN vs control 2.0 fold down regulated miRNAs							
		Fold chang	ForeGround		eGround-BackGro		Normalize
ID	Name	VAN vs control	control	VAN	control	VAN	control
11052	hsa-miR-31-5p	0.36367	363	171	307.5	98.5	1.33261
145843	hsa-miR-330-5p	0.21221	107	78.5	53.5	10	0.23185
42524	hsa-miR-21-3p	0.33596	110	85.5	49	14.5	0.21235
145641	hsa-miR-369-5p	0.36642	147.5	98.5	94.5	30.5	0.40953
148327	hsa-miR-3651	0.44724	124.5	94	66	26	0.28602
169199	hsa-miR-4518	0.38616	354.5	170.5	294	100	1.27411
42496	hsa-miR-181c-5p	0.45987	93.5	85.5	39.5	16	0.17118
10306	hsa-miR-146b-5p	0.47528	457.5	238	402.5	168.5	1.74431
17882	hsa-miR-20b-3p	0.46893	101.5	88	46	19	0.19935
10943	hsa-miR-136-5p	0.48216	4201	1835	4146.5	1761	17.9697
146098	hsv1-miR-H5-3p	0.39132	168.5	111.5	117.5	40.5	0.50921
146161	hsa-miR-2115-3p	0.48807	103.5	93.5	53.5	23	0.23185
145693	hsa-miR-92a-3p	0.49361	154	112.5	103.5	45	0.44854
10937	hsa-miR-132-3p	0.44583	159.5	113.5	109.5	43	0.47454
46944	hsa-miR-1297	0.4961	109.5	96	59.5	26	0.25785
145643	hsa-miR-382-5p	0.40409	198	127.5	147.5	52.5	0.63922
169159	hsa-miR-4521	0.44325	250	146	198.5	77.5	0.86024
19585	hsa-miR-148b-3p	0.40137	107.5	86.5	49.5	17.5	0.21452
145844	hsa-miR-374a-5p	0.34754	112.5	88.5	49	15	0.21235
145859	hsa-miR-33a-5p	0.47519	772.5	369.5	706	295.5	3.05959
31867	hsa-miR-145-3p	0.33962	111	87.5	58.5	17.5	0.25352
145634	hsa-miR-132-5p	0.13723	100.5	79	45.5	5.5	0.19718

46320	hsa-miR-31-3p	0.14995	106.5	79.5	53	7	0.22969
10925	hsa-miR-10b-5p	0.3211	104	85	49.5	14	0.21452
42839	hsa-miR-135a-5p	0.4953	643	328.5	584.5	255	2.53304
46737	hsa-miR-1265	0.46444	86.5	88.5	33	13.5	0.14301
13140	hsa-miR-138-5p	0.36611	529.5	226.5	476	153.5	2.06284
11013	hsa-miR-181a-3p	0.45138	96	90.5	41.5	16.5	0.17985
29802	hsa-miR-144-3p	0.49242	134	113.5	83	36	0.3597
145749	hsa-miR-137	0.36973	191	121.5	130.5	42.5	0.56555
145746	hsa-let-7i-3p	0.20787	90.5	78.5	35.5	6.5	0.15385
10986	hsa-miR-193a-3p	0.47502	182	119.5	119.5	50	0.51788
42451	hsa-miR-139-3p	0.21209	99.5	78	45.5	8.5	0.19718
147997	hsa-miR-3934-5p	0.28383	112	87.5	54	13.5	0.23402
148559	hsa-miR-411-3p	0.35913	101.5	88.5	49	15.5	0.21235
46215	hsa-miR-1301-3p	0.44647	102	100	44.5	17.5	0.19285
42950	hsa-miR-24-2-5p	0.35061	87	83	34	10.5	0.14735
11053	hsa-miR-32-5p	0.48871	131.5	106	75.5	32.5	0.32719
42599	hsa-miR-153-3p	0.44844	457	245.5	400	158	1.73348
148645	hsa-miR-129-5p	0.39661	170.5	141	114.5	40	0.49621
168887	hsa-miR-5089-5p	0.35017	1250.5	504.5	1191.5	367.5	5.1636
169271	hsa-miR-4784	0.26246	152	161.5	93	21.5	0.40303
28950	hsa-miR-455-3p	0.35927	98.5	90.5	39.5	12.5	0.17118
27544	hsa-miR-363-5p	0.25015	119	87	59	13	0.25569
147771	hsa-miR-4328	0.06342	142.5	75	89.5	5	0.38787
13147	hsa-miR-96-5p	0.18639	88.5	96	33.5	5.5	0.14518
29490	hsa-miR-7-5p	0.35317	454	227	397	123.5	1.72048
17280	hsa-miR-15b-5p	0.39736	225.5	142	170	59.5	0.73673
42887	hsa-miR-331-3p	0.34779	196.5	136	142	43.5	0.61538
168871	hsa-miR-151a-5p/hs	0.41448	120	98	63	23	0.27302
168980	hsa-miR-4324	0.48296	303	177	248	105.5	1.07476
42866	hsa-miR-451a	0.40891	398.5	204	341.5	123	1.47996
13171	hsa-miR-429	0.35779	141.5	110	82.5	26	0.35753
42532	hsa-miR-22-5p	0.46161	108.5	100.5	45.5	18.5	0.19718
148418	hsa-miR-3607-5p	0.3469	99	92	36	11	0.15601
168864	hsa-miR-320d	0.47009	124.5	108.5	64	26.5	0.27736
17917	hsa-miR-873-5p	0.36762	107.5	85	52.5	17	0.22752
17888	hsa-let-7a-3p	0.42823	116.5	90	57	21.5	0.24702
17377	hsa-miR-600	0.41134	92	77.5	34.5	12.5	0.14951
42739	hsa-miR-339-5p	0.27615	92.5	79	37	9	0.16035
13177	hsa-miR-143-3p	0.19536	781.5	194	723.5	124.5	3.13543
11078	hsa-miR-365a-3p/hs	0.34101	197.5	126	136.5	41	0.59155
10987	hsa-miR-193b-3p	0.42574	102	98.5	40	15	0.17335
146049	hsa-miR-28-5p	0.4325	129	95	73.5	28	0.31853
168925	hsa-miR-1273g-3p	0.43592	412.5	204.5	355.5	136.5	1.54063
