# Supplementary material for: MBD2 upregulates miR-301a-5p to induce kidney cell apoptosis during vancomycin-induced AKI
Source: Cell Death Dis. 2017 Oct 12;8(10):e3120–. doi: 10.1038/cddis.2017.509 (PMC5682674; doi:10.1038/cddis.2017.509)
Supplement: Supplementary Table 2 [file cddis2017509x2.pdf]

Condition pairs: MBD2 siRNA with VAN vs Control# Fold Change cut-off: 2.0

Column "ID": array ID of the probes, each miRNA always has its unique probe, but some miRNAs may have two different probes.
Column "Name": the name of each miRNA.
Column "Fold change": the ratio of normalized intensities between two conditions (use normalized data, ratio scale).
Column "ForeGround": the foreground intensity of each probe.
Column "ForeGround-BackGround": the signal of the probe after background correction.
Column "Normalized": the normalized ratio of the microRNA. Median Normalization Method was adopted.

NOTE: The low intensity differentially expressed miRNAs are filtered in the following list (miRNAs that ForeGround-BackGround intensities < 30 in two samples are filtered). If you

MBD2 siRNA with VAN vs Control 2.0 fold up regulated miRNAs								
		Fold chang	ForeGround		eGround-BackGrou		Normalized	
ID	Name	MBD2 siRNA with VAN vs Control	control	MBD2 siRNA with VAN	control	MBD2 siRNA with VAN	con trol	MBD2 siRNA with VAN
17928	hsa-miR-181a-2-3p	5.54823	66.5	124.5	13	64	0.1	0.31258
42696	hsa-miR-943	9.74036	61.5	129	7	60.5	0	0.29548
42795	kshv-miR-K12-3-5p	4.20058	62.5	107.5	11	41	0	0.20024
146117	hsv1-miR-H6-3p	11.2698	56.5	107.5	4.5	45	0	0.21978
145838	hsa-miR-125b-1-3p	4.2575	69.5	102.5	9	34	0	0.16606
145678	hsa-miR-150-5p	3.20417	77	141.5	25.5	72.5	0.1	0.35409
147165	hsa-let-7b-5p	2.06643	695	1240	640	1173.5	2.8	5.73138
168640	hsa-miR-4475	4.93541	115	320	58	254	0.3	1.24054
169170	hsa-miR-4472	4.62657	64	104	9.5	39	0	0.19048
169043	hsa-miR-4462	6.64921	60.5	121.5	10	59	0	0.28816
169358	hsa-miR-4417	4.50794	59.5	91.5	7.5	30	0	0.14652
147595	hsa-miR-3178	3.81306	149.5	394	96.5	326.5	0.4	1.59463
27565	hsa-miR-423-5p	3.57903	215.5	590	165	524	0.7	2.55922
147767	hsa-miR-4279	3.59449	109	217	47.5	151.5	0.2	0.73993
148682	hsa-miR-483-3p	3.03248	130.5	272.5	76	204.5	0.3	0.99878
169282	hsa-miR-4290	3.52421	124.5	248.5	59	184.5	0.3	0.9011
169034	hsa-miR-642b-5p	3.85461	91	182.5	34.5	118	0.1	0.57631
27568	hsa-miR-744-5p	3.30888	137	316	86	252.5	0.4	1.23321
42832	hsa-miR-638	4.7709	61.5	128	15	63.5	0.1	0.31013
146090	hsv1-miR-H7-3p	3.06235	168.5	391.5	118.5	322	0.5	1.57265
147938	hsa-miR-4287	5.47392	66	122	10.5	51	0	0.24908
148687	hsa-miR-1908-5p	3.77253	111.5	262.5	59	197.5	0.3	0.96459
168572	hsa-miR-4507	5.63492	64	102	7	35	0	0.17094
169320	hsa-miR-4468	2.93016	117.5	226	60	156	0.3	0.7619
169319	hsa-miR-3136-3p	2.47937	91.5	157.5	42.5	93.5	0.2	0.45665
42502	hsa-miR-204-3p	5.12509	114	354	63	286.5	0.3	1.39927
46789	hsa-miR-513b-5p	2.67137	64	103	13.5	32	0.1	0.15629
168919	hsa-miR-4456	3.65328	248	696.5	194.5	630.5	0.8	3.07937
168661	hsa-miR-4531	6.38624	58.5	101	6	34	0	0.16606
168776	hsa-miR-4795-3p	6.36537	105	371	54	305	0.2	1.48962

168998	hsa-miR-4508	13.2734	55	120.5	4.5	53	0	0.25885
42782	hcmv-miR-UL148D	3.11048	69	102	12.5	34.5	0.1	0.1685
46336	hsa-miR-1284	7.2034	107	374.5	48.5	310	0.2	1.51404
42581	hsa-miR-513a-5p	6.86674	76.5	197	21.5	131	0.1	0.6398
168843	hsa-miR-5694	4.01206	69.5	113	12.5	44.5	0.1	0.21734
168995	hsa-miR-4791	2.13811	164.5	276.5	107	203	0.5	0.99145
169050	hsa-miR-4787-5p	6.82098	189.5	831	124	750.5	0.5	3.66545
146165	hsa-miR-1973	2.14127	83	106.5	20	38	0.1	0.18559
168727	hsa-miR-4426	3.94444	61.5	105.5	10	35	0	0.17094
168971	hsa-miR-4449	6.38624	63.5	132	12	68	0.1	0.33211
169087	hsa-miR-149-3p	4.59142	63.5	119.5	13.5	55	0.1	0.26862
146196	hsa-miR-711	4.92193	104	282.5	49	214	0.2	1.04518
148085	hsa-miR-3687	18.7831	61	189	7.5	125	0	0.6105
168765	hsa-miR-4448	5.68859	65	119.5	10.5	53	0	0.25885
168928	hsa-miR-4431	2.04453	110	167	56.5	102.5	0.2	0.50061
169079	hsa-miR-4667-5p	2.79899	118.5	215.5	61	151.5	0.3	0.73993
169399	hsa-miR-4750-5p	4.08654	113	273	57.5	208.5	0.2	1.01832
145768	hsa-miR-665	3.38095	112	246.5	59.5	178.5	0.3	0.87179
147604	hsa-miR-4285	4.71079	80.5	173	25	104.5	0.1	0.51038
147804	hsv1-miR-H17	10.5856	64	203	14	131.5	0.1	0.64225
148263	hsa-miR-1273e	4.8836	64.5	118	12	52	0.1	0.25397
168893	hsa-miR-4505	4.93541	67	135.5	14.5	63.5	0.1	0.31013
169179	hsa-miR-4650-5p	3.94444	88.5	174.5	29	101.5	0.1	0.49573
42929	hsa-miR-25-5p	2.46097	78	118.5	24.5	53.5	0.1	0.26129
148032	hsa-miR-3685	2.01587	88.5	128.5	35.5	63.5	0.2	0.31013
17488	kshv-miR-K12-6-3p	7.1539	81.5	212.5	23	146	0.1	0.71306
168921	hsa-miR-4718	6.41514	62.5	99	6.5	37	0	0.18071
169285	hsa-miR-4467	2.90572	308	703	249	642	1.1	3.13553
169379	hsa-miR-4694-3p	3.9914	62	109	12	42.5	0.1	0.20757
169232	hsa-miR-3156-3p	2.07365	114	177.5	62.5	115	0.3	0.56166
169395	hsa-miR-4484	2.03347	106.5	147	46	83	0.2	0.40537
146072	hsa-miR-1469	5.14187	83	180.5	24	109.5	0.1	0.5348
147942	hsa-miR-4268	6.14206	86.5	230.5	30	163.5	0.1	0.79853
148622	hsa-miR-877-3p	4.09053	70.5	114.5	13.5	49	0.1	0.23932
169375	hsa-miR-660-3p	3.92921	107.5	258.5	55.5	193.5	0.2	0.94505
169189	hsa-miR-4795-5p	3.28704	65	106.5	12	35	0.1	0.17094
147631	hsa-miR-4258	6.1045	60.5	94.5	6	32.5	0	0.15873
148156	hsa-miR-3686	2.67925	158.5	315.5	106	252	0.5	1.23077
168941	hsa-miR-4501	2.77989	91	153.5	37.5	92.5	0.2	0.45177
169116	hsa-miR-4788	4.38272	65.5	115.5	13.5	52.5	0.1	0.25641
42702	hsa-miR-30c-1-3p	3.87095	78	142.5	23	79	0.1	0.38584
168638	hsa-miR-4530	7.34418	88	258.5	30	195.5	0.1	0.95482
169024	hsa-miR-3960	3.2052	371	949.5	311	884.5	1.3	4.3199
169028	hsa-miR-4708-3p	4.14485	177	504	119.5	439.5	0.5	2.14652
13137	hsa-miR-518e-5p/hs	4.71284	73	114	11	46	0	0.22466
42458	hcmv-miR-US25-1-3	9.48545	70.5	125	6	50.5	0	0.24664
148678	hsa-miR-301a-5p	2.49986	87	127	27.5	61	0.1	0.29792
147738	hsv2-miR-H20	2.56133	93.5	145.5	33	75	0.1	0.3663
168935	hsa-miR-4687-3p	4.22619	73	125.5	16	60	0.1	0.29304
168672	hsa-miR-1587	12.1714	66.5	149	7.5	81	0	0.3956
17668	hsa-miR-552-3p	7.3254	65	131.5	11	71.5	0	0.34921

147790	hsv2-miR-H7-3p	4.50794	80.5	169	27	108	0.1	0.52747
168978	hsa-miR-371b-5p	3.26375	178	418	125	362	0.5	1.76801
169228	hsa-miR-4698	2.3291	82.5	122	30	62	0.1	0.30281
169110	hsa-miR-4497	4.81806	330.5	1247.5	278	1188.5	1.2	5.80464
169313	hsa-miR-4800-3p	5.95692	293.5	1322	238	1258	1	6.14408
169155	hsa-miR-4480	2.39484	73	97	16	34	0.1	0.16606
169388	hsa-miR-663a	7.67758	66	116.5	8	54.5	0	0.26618
147667	hsa-miR-3182	2.96772	71.5	110	15	39.5	0.1	0.19292
168944	hsa-miR-4707-5p	4.30669	71.5	118.5	14	53.5	0.1	0.26129
168660	hsa-miR-5000-5p	11.2698	61.5	120.5	5.5	55	0	0.26862

MBD2 siRNA with VAN vs Control 2.0 fold down regulated miRNAs

		Fold chang	ForeGround		eGround-BackGrou		Normalized	
ID	Name	MBD2 siRNA with VAN vs Control	control	MBD2 siRNA with VAN	control	MBD2 siRNA with VAN	con trol	MBD2 siRNA with VAN
145693	hsa-miR-92a-3p	0.41922	154	107	103.5	38.5	0.4	0.18803
145643	hsa-miR-382-5p	0.4699	198	133	147.5	61.5	0.6	0.30037
145844	hsa-miR-374a-5p	0.1955	112.5	84.5	49	8.5	0.2	0.04151
145634	hsa-miR-132-5p	0.37153	100.5	81.5	45.5	15	0.2	0.07326
46320	hsa-miR-31-3p	0.27643	106.5	81.5	53	13	0.2	0.06349
10925	hsa-miR-10b-5p	0.35289	104	78.5	49.5	15.5	0.2	0.0757
13140	hsa-miR-138-5p	0.40841	529.5	243.5	476	172.5	2.1	0.84249
145746	hsa-let-7i-3p	0.42857	90.5	81.5	35.5	13.5	0.2	0.06593
11005	hsa-miR-204-5p	0.38957	92	79.5	40.5	14	0.2	0.06838
42451	hsa-miR-139-3p	0.19815	99.5	72	45.5	8	0.2	0.03907
147997	hsa-miR-3934-5p	0.32349	112	81	54	15.5	0.2	0.0757
148559	hsa-miR-411-3p	0.35649	101.5	81	49	15.5	0.2	0.0757
28950	hsa-miR-455-3p	0.31384	98.5	81.5	39.5	11	0.2	0.05372
27544	hsa-miR-363-5p	0.48709	119	93.5	59	25.5	0.3	0.12454
147771	hsa-miR-4328	0.20147	142.5	77	89.5	16	0.4	0.07814
168915	hsa-miR-4780	0.47584	127.5	93	67.5	28.5	0.3	0.13919
13147	hsa-miR-96-5p	0.47098	88.5	78.5	33.5	14	0.1	0.06838
46918	hsa-miR-375	0.13744	95.5	67	41	5	0.2	0.02442
29490	hsa-miR-7-5p	0.43149	454	216.5	397	152	1.7	0.74237
13171	hsa-miR-429	0.36883	141.5	98	82.5	27	0.4	0.13187
42532	hsa-miR-22-5p	0.34676	108.5	86	45.5	14	0.2	0.06838
10952	hsa-miR-146a-5p	0.3734	391.5	172.5	332	110	1.4	0.53724
13177	hsa-miR-143-3p	0.23755	781.5	219.5	723.5	152.5	3.1	0.74481
145640	hsa-miR-328-3p	0.01708	90	63	33	0.5	0.1	0.00244
13133	hsa-miR-520a-5p	0.40833	91	85	34.5	12.5	0.1	0.06105
46801	hsa-miR-106a-5p	0.49705	287.5	172.5	229	101	1	0.49328
10987	hsa-miR-193b-3p	0.45079	102	87	40	16	0.2	0.07814
146049	hsa-miR-28-5p	0.253	129	79.5	73.5	16.5	0.3	0.08059
