# Supplementary material for: MBD2 upregulates miR-301a-5p to induce kidney cell apoptosis during vancomycin-induced AKI
Source: Cell Death Dis. 2017 Oct 12;8(10):e3120–. doi: 10.1038/cddis.2017.509 (PMC5682674; doi:10.1038/cddis.2017.509)
Supplement: Supplementary Table 3 [file cddis2017509x3.pdf]

Condition pairs: MBD2 siRNA with VAN vs VAN
Fold Change cut-off: 2.0

Column "ID": array ID of the probes, each miRNA always has its unique probe, but some miRNAs may have two different probes.
Column "Name": the name of each miRNA.
Column "Fold change": the ratio of normalized intensities between two conditions (use normalized data, ratio scale).
Column "ForeGround": the foreground intensity of each probe.
Column "ForeGround-BackGround": the signal of the probe after background correction.
Column "Normalized": the normalized ratio of the microRNA. Median Normalization Method was adopted.

NOTE: The low intensity differentially expressed miRNAs are filtered in the following list

MBD2 siRNA with VAN vs VAN 2.0 fold up regulated miRNAs

		Fold chang	ForeGround		eGround-BackGrou		Normalized	
ID	Name	siRNA with VAN vs VAN	VAN	MBD2 siRNA with VAN	VAN	MBD2 siRNA with VAN	VAN	MBD2 siRNA with VAN
42696	hsa-miR-943	2.4513	96	129	24.5	60.5	0.121	0.29548
169043	hsa-miR-4462	3.16583	85	121.5	18.5	59	0.091	0.28816
169358	hsa-miR-4417	4.25432	75.5	91.5	7	30	0.034	0.14652
145641	hsa-miR-369-5p	2.18063	98.5	136	30.5	67	0.15	0.32723
169199	hsa-miR-4518	2.32782	170.5	300	100	234.5	0.492	1.1453
42496	hsa-miR-181c-5p	2.07841	85.5	99	16	33.5	0.079	0.16361
17882	hsa-miR-20b-3p	2.01147	88	104.5	19	38.5	0.093	0.18803
169159	hsa-miR-4521	2.0686	146	228	77.5	161.5	0.381	0.78877
19585	hsa-miR-148b-3p	2.49587	86.5	114	17.5	44	0.086	0.2149
168843	hsa-miR-5694	3.53392	86.5	113	12.5	44.5	0.062	0.21734
168995	hsa-miR-4791	2.03548	173	276.5	99	203	0.487	0.99145
31867	hsa-miR-145-3p	3.17656	87.5	122	17.5	56	0.086	0.2735
42542	hsa-miR-589-5p	2.34886	254	493	177.5	420	0.873	2.05128
168887	hsa-miR-5089-5p	2.74032	504.5	1081	367.5	1014.5	1.808	4.95482
169271	hsa-miR-4784	4.47858	161.5	164.5	21.5	97	0.106	0.47375
168925	hsa-miR-1273g-3p	2.02535	204.5	348	136.5	278.5	0.672	1.3602

MBD2 siRNA with VAN vs VAN 2.0 fold down regulated miRNAs

		Fold chang	ForeGround		eGround-BackGrou		Normalized	
ID	Name	siRNA with VAN vs VAN	VAN	MBD2 siRNA with VAN	VAN	MBD2 siRNA with VAN	VAN	MBD2 siRNA with VAN
168861	hsa-miR-4754	0.30212	100.5	76	34.5	10.5	0.17	0.05128
168954	hsa-miR-5580-5p	0.45874	133	96.5	66	30.5	0.325	0.14896
147938	hsa-miR-4287	0.42543	188.5	122	119	51	0.585	0.24908
148284	hsa-miR-208b-3p	0.35989	446.5	201	376.5	136.5	1.852	0.66667
146158	hsa-miR-3202	0.40561	303	163.5	232.5	95	1.144	0.46398
17529	hcmv-miR-US25-2-3	0.4467	101	75.5	30	13.5	0.148	0.06593
42869	hsa-miR-936	0.12073	107	69	37	4.5	0.182	0.02198
169181	hsa-miR-5191	0.06812	117	65	51	3.5	0.251	0.01709

147651	hsa-miR-3123	0.42208	142.5	96	63.5	27	0.312	0.13187
147930	hsa-miR-3144-3p	0.42803	123.5	84	54.5	23.5	0.268	0.11477
169012	hsa-miR-4711-3p	0.35266	107.5	78.5	38	13.5	0.187	0.06593
169137	hsa-miR-4524b-5p	0.37685	139.5	84.5	54	20.5	0.266	0.10012
148678	hsa-miR-301a-5p	0.38204	236.5	127	158.5	61	0.78	0.29792
168586	hsa-miR-34a-5p	0.37669	285	147	209.5	79.5	1.031	0.38828
42673	hsa-miR-337-3p	0.01249	1858.5	82.5	1788.5	22.5	8.8	0.10989
23767	hsa-miR-759	0.07755	103.5	65	32	2.5	0.157	0.01221
42661	hsa-miR-492	0.23706	103.5	74	33.5	8	0.165	0.03907
168841	hsa-miR-5588-3p	0.43086	187	116	117.5	51	0.578	0.24908
46731	hsa-miR-4657	0.48531	405	227.5	337.5	165	1.661	0.80586
169212	hsa-miR-514a-5p	0.31084	122.5	82	49.5	15.5	0.244	0.0757
46408	hsa-miR-1322	0.06056	268.5	83	188.5	11.5	0.927	0.05617
